# Supplementary material for: Simulation applications to support teaching and research in epidemiological dynamics
Source: BMC Med Educ. 2022 Aug 20;22:632. doi: 10.1186/s12909-022-03674-3 (PMC9391658; doi:10.1186/s12909-022-03674-3)
Supplement: Supplementary file 1 — Additional file 1: Supplementary online file. This file contains the following Appendices: A. Simulation Exercises; B. Boxcar Models; C. Waiting Times in SIRS ABMs. [file 12909_2022_3674_MOESM1_ESM.pdf]

Appendices

A. Simulation Exercises

The number of these exercises correspond to the material in the subsection numbers of Results Sections 3.1-3.6

Exercise 1.

By varying the value of κ between 1 and 8, with other parameter values fixed as in Fig. 3, use the SIRS RAMP to:

1.1. explore the relationship between the time t^* and value $I(t^*)/N(t^*)$ of maximum prevalence

1.2. explore the relationship between the time t^* of maximum prevalence and the time t_1 when prevalence first rises above 0.1%

1.3 explore the relationship between the time t^* of maximum prevalence and the time $t_2 - t_1$ it takes for prevalence to drop below 0.1% at time t_2, and note the value of $R_{\text{eff}}(t_2)$ in each case

1.4 plot the proportion of individuals that escape infection in each case as a function of your computed value for R_0 as a function of the selected value for κ.

Exercise 2.

Plot (either as a series of 1-D curves or a 2-D surface) the relationship between the ratio of peak to endemic prevalence as a function of R_0 (through manipulation of the value κ_0) and the mean residence time $\bar{T}^{\text{R}} = 1/\rho_{\text{RS}}$

Exercise 3.

By varying the strength of adaptive behavior through changes in the prevalence values of the behavioral switch parameter P_κ estimate the period of endemic oscillations that dampen over a five-year simulation interval as a function of the values of P_κ and the mean waiting time $\bar{T}^{\text{R}} = 1/\rho_{\text{RS}}$ for combinations of these parameters where dampened oscillatory behavior is evident. (Note: this will require identification of local maxima in the prevalence curve over the five year simulation interval and computing the average time between consecutive local maxima over the five year period for each of selected pair of parameter values.)

Exercise 4.

4.1. By varying the value of κ_0 between 1 and 8, with other parameter values fixed as in the stochastic SIRS RAMP depicted in Fig. 3, construct a histogram of times at which the prevalence $I(t)$ becomes 0, as shown in Fig. 13. Note for each value κ_0 at least 1000 runs should be made, as depicted in Fig. 13. Using the calculations shown in Eq. 22 and 25, evaluate the outbreak probability for the set of model parameters used in the simulations. In addition, evaluate the proportion of simulations which resulted in a major outbreak, as estimation of the outbreak probability. Plot the two results as function of κ_0.

4.2. From from the runs made Ex. 4.1, plot the mean $\pm$ proportion (on the vertical axis) of runs in the major outbreak component and its standard deviation (assuming a binomial distribution for the mean and variance) as a function of the value of R_0 (horizontal axis) that corresponds to the eight values of κ involved. Over lay this with a plot of Eq. 25 and comment on the fit.

4.3 (Advanced exercise) For $\kappa_0 = 4.0$ and $\rho_{\text{RS}} = 0.03$, with other parameter values as in the stochastic SIRS RAMP depicted in Fig. 3, carry out 1000 simulations over the interval of time $[0, 1200]$. Select at random 10 of the simulations that constitutes a major outbreak (you might as well take the first 10 since the are randomly organized themselves), and plot a histogram of the 1000 values between $t = 201$ and $t = 1200$ for each of these 10 simulations. Compare normalized versus of these 10 histograms among themselves (i.e., reduce bins from numbers to proportions so the area under the histogram is 1—i.e., it is an empirical probability distribution). Also compare this histograms to normalized histograms of the values across all simulations that are part of the major outbreak at time $t = 1200$. Repeat this exercise for all values at time $t = 1000$. What do you notice. What does this tell you about the *ergodicity* of the SIRS stochastic model

Exercise 5.

Come up with creative ways to explore the complexities of including treatment as a consequence of the rate at which individuals are treated, limitations on the number of individuals that can be treated at anyone time, and assumed effects of treatment. These effects include reducing mortality in the population as a whole and making assumptions about mortality rates for individuals that are treated.

Exercise 6.

Come up with creative ways to explore the complexities of vaccination programs as a consequence of starting dates, vaccination rates, and limitations on the number of regimens available—either in absolute times or in monthly tranches.

Solution to Exercise 4.1

In Figure A.1 (left panel) we report the histograms of times at which the prevalence $I(t)$ became 0, for values of κ_0 varying from 1 to 8 and with other parameter value fixed as in the stochastic SIRS RAMP depicted in Fig. 3. In Figure A.1 (right panel) we show the outbreak probability values plotted in red when computed from the model parameters using Eq. 22 (to compute R_0) and Eq. 25 and plotted in blue when calculated using the proportion of simulations for which we observed a major outbreak, as shown in Figure 13.

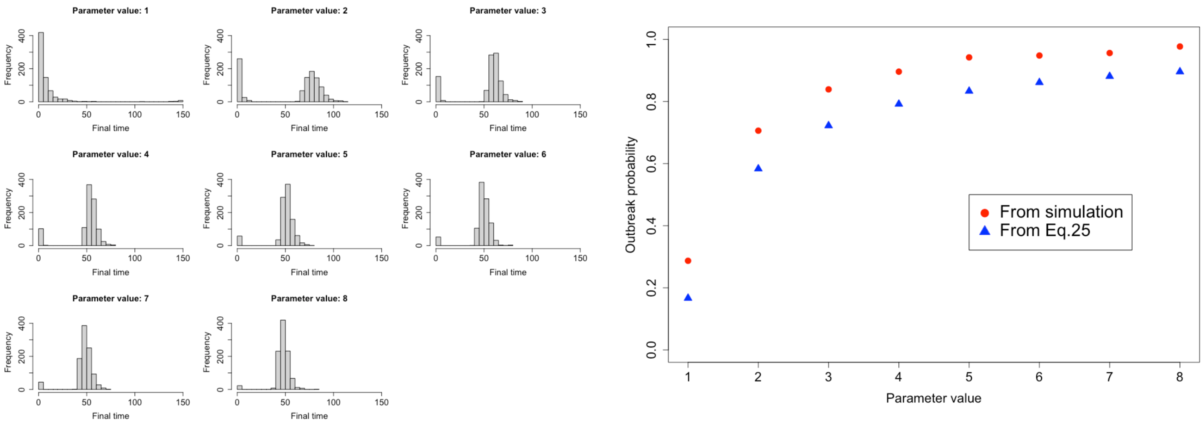

Figure A.1: Solution of Exercise 4.1. On the left we show the histograms of times at which the prevalence $I(t)$ became 0, as a function of the value of the parameter value κ_0. On the right panel, we show the estimation of the outbreak probability using two different approaches for its calculation (noting for the parameters used in our simulation that $R_0 = 1.2\kappa_0$).

B. Boxcar Models

The exponential transfer distribution of Eq. 1.1 in Box 1 implies a maximum transfer rate of $1/\rho$ at $t = 0$ of a flow through disease class X. This is unrealistic since one would expect the maximum transfer rate not to be the moment of entering X but rather around the some mean period of time in X. This can be remedied by subdividing X into K sub-compartments x_k, $k = 1, ..., K$ each of which is traversed at a rate $K\rho$. In this case we obtain a *boxcar transfer process through disease class X*, modeled by the following system of equations

$$\begin{aligned} \frac{dx_1}{dt} &= -K\rho x_1 \quad x_1 = X_0 \\ \frac{dx_k}{dt} &= K\rho\left(x_{k-1} - x_k\right), \quad k = 2, ..., K-1 \\ \frac{dx_K}{dt} &= K\rho x_{K-1} \end{aligned} \tag{B.1}$$

The solution X(t) to this systems of equation is known to be 1 minus the Erlang distribution multiplied by X_0. Specifically,

$$X(t) = X_0 \sum_{k=0}^{K-1} \frac{e^{-K\rho t}(K\rho t)^k}{k!} \tag{B.2}$$

This also implies in the context of individuals passing through X—that is, through all K boxcars that constitute X—that

$$\text{Prob[Leave X by time } t] \equiv F(t; K, K\rho) = \begin{cases} 1 - \sum_{k=0}^{K-1} \frac{e^{-K\rho t}(K\rho t)^k}{k!} & t \geq 0 \\ 0 & \text{otherwise} \end{cases} \tag{B.3}$$

with corresponding Erlang probability density function (shape parameter K, scale parameter $K\rho$

$$f(t; K, K\rho) = \frac{dF}{dt} = \begin{cases} \frac{(K\rho)^K \, t^{K-1} \, e^{-K\rho t}}{(K-1)!} & t \geq 0 \\ 0 & \text{otherwise} \end{cases} \tag{B.4}$$

Thus, by computing $\int_0^\infty t f(t) dt$, the mean time $\bar{T}^{\mathrm{X}}$ each individual spends in class X is

$$\underline{\text{Mean time spent in class X:}} \qquad \bar{T}^{\mathrm{X}} = \frac{1}{\rho} \tag{B.5}$$

The mode is no longer at 0, but now is somewhat below the mean with a value $\frac{K-1}{K\rho}$. The variance of this distribution is $\frac{1}{K\rho^2}$ and as $K \to \infty$, all individuals spend the same amount of time $\bar{T}^X = 1/\rho$ in X.

C. Waiting Times in SIRS ABMs

Suppose the per capita outflow rate of individuals from disease class X is an increasing function of how long these individuals have been in X. We investigate the consequences of such an assumption by considering the exit distribution of X_0 individuals who entered disease class X at time 0 under the assumption that their per capita rate of outflow from X is given by the function $\rho(t) = \rho_0 t^k$, for constants $\rho_0 > 0$ and $k \geq 0$. This process can be described by the differential equation (compare with equations in Box 1)

$$\frac{dX}{dt} = -\rho_0 t^k X, \qquad X(0) = 1 \tag{C.6}$$

$$\iff X(t) = e^{-\rho_0 t^{k+1}/(k+1)} \tag{C.7}$$

This also implies that

$$\text{Prob[Leave X by time } t] \equiv F(t) = \begin{cases} 1 - e^{-\rho_0 t^{k+1}/(k+1)}) & t \geq 0 \\ 0 & \text{otherwise} \end{cases} \tag{C.8}$$

with corresponding probability density function

$$f(t) = \frac{dF}{dt} = \begin{cases} \rho_0 t^k e^{-\rho_0 t^{k+1}/(k+1)} & t \geq 0 \\ 0 & \text{otherwise} \end{cases} \tag{C.9}$$

Thus, by computing $\int_0^\infty t f(t) dt$, the mean time $\bar{T}^{\mathrm{X}}$ each individual spends in class X is in terms of the Gamma function $\Gamma(\cdot)$ (which is a generalization of the factorial function to real numbers)

$$\underline{\text{Mean time spent in class X:}} \qquad \bar{T}^{\mathrm{X}}(\rho_0, k) = \left(\frac{k+1}{\rho_0}\right)^{1/(k+1)} \Gamma\left(\frac{k+2}{k+1}\right) \tag{C.10}$$

Thus it follows that

$$\bar{T}^{\mathrm{X}}(\rho_0, 0) = \frac{1}{\rho_0}, \;\; \bar{T}^{\mathrm{X}}(\rho_0, 1) \approx \frac{1.253}{\sqrt{\rho_0}}, \text{ and } \; T^{\mathrm{X}}(\rho_0, 2) \approx \frac{1.288}{\sqrt[3]{\rho_0}} \tag{C.11}$$

and that $\bar{T}^{\mathrm{X}}(a, 3) \approx \frac{1.282}{\sqrt[4]{\rho_0}}$, $\bar{T}^{\mathrm{X}}(a, 4) \approx \frac{1.267}{\sqrt[5]{\rho_0}}$, and $\bar{T}^{\mathrm{X}}(a, 9) \approx \frac{1.041}{\sqrt[10]{\rho_0}}$. More generally, as a mathematical curiosity, it appears from numerical experiments that $\bar{T}^{\mathrm{X}}(a, k) \approx \frac{1}{\sqrt[k]{\rho_0}}$ as $k \to \infty$.
